# Producing 3D Biomimetic Nanomaterials for Musculoskeletal System Regeneration

**DOI:** 10.3389/fbioe.2018.00128

**Published:** 2018-09-20

**Authors:** Ignasi Casanellas, Andrea García-Lizarribar, Anna Lagunas, Josep Samitier

**Affiliations:** ^1^Nanobioengineering Group, Institute for Bioengineering of Catalonia, The Barcelona Institute of Science and Technology, Barcelona, Spain; ^2^Department of Electronics and Biomedical Engineering, University of Barcelona, Barcelona, Spain; ^3^Networking Biomedical Research Center (CIBER), Madrid, Spain

**Keywords:** nanofiber, 3D printing, stimuli-responsive, musculoskeletal, regeneration, scaffold, tissue engineering

## Abstract

The human musculoskeletal system is comprised mainly of connective tissues such as cartilage, tendon, ligaments, skeletal muscle, and skeletal bone. These tissues support the structure of the body, hold and protect the organs, and are responsible of movement. Since it is subjected to continuous strain, the musculoskeletal system is prone to injury by excessive loading forces or aging, whereas currently available treatments are usually invasive and not always effective. Most of the musculoskeletal injuries require surgical intervention facing a limited post-surgery tissue regeneration, especially for widespread lesions. Therefore, many tissue engineering approaches have been developed tackling musculoskeletal tissue regeneration. Materials are designed to meet the chemical and mechanical requirements of the native tissue three-dimensional (3D) environment, thus facilitating implant integration while providing a good reabsorption rate. With biological systems operating at the nanoscale, nanoengineered materials have been developed to support and promote regeneration at the interprotein communication level. Such materials call for a great precision and architectural control in the production process fostering the development of new fabrication techniques. In this mini review, we would like to summarize the most recent advances in 3D nanoengineered biomaterials for musculoskeletal tissue regeneration, with especial emphasis on the different techniques used to produce them.

## Introduction

The musculoskeletal system comprises connective tissues such as cartilage, tendon, ligaments, skeletal muscle, and skeletal bone. It provides shape and support to the body and confers the ability to move. Musculoskeletal disorders (MSDs) are injuries and/or pain affecting the musculoskeletal system. They are one of the main causes of disability worldwide with an increasing number of diagnosed cases each year and an estimated cost of $125 billion per year (Storheim and Zwart, 2014). MSDs are caused by mechanical loading including heavy loads, repetitive motions or maintained static positions. Common MSDs include tendinitis, carpal tunnel syndrome, osteoarthritis, rheumatoid arthritis, fibromyalgia and bone fractures, among others. These conditions frequently entail significant loss of tissue, and treatment of such severe and widespread musculoskeletal lesions normally requires surgical intervention.

The existing surgical techniques used to repair the musculoskeletal system are hampered by the limited accessibility, amount and quality of materials used, such as grafts. Therefore, tissue engineering and regenerative medicine are postulated as a reliable and promising option to overcome this clinical need. Implants that induce tissue formation at the site of injury have been designed and produced (Smith and Grande, 2015). An implant for tissue regeneration has a primary structure or scaffold which mimics host tissue biomechanics to promote integration. The scaffold must be biocompatible, low immunogenic, allow cell infiltration, nutrient and waste exchange, stand sterilization procedures and be easy to handle during surgery. The engineered scaffolds can be loaded with cells and can be biodegradable to enable the replacement of the scaffold by the host tissue. If biodegradable, the scaffold degradation time should meet the growing time of the newly formed tissue.

The musculoskeletal system comprises tissues with distinctive characteristics ranging from cortical bone, which is a hard (elastic modulus of 16–23 GPa), highly vascularized tissue with self-healing capabilities, to cartilage that is a soft (elastic modulus of 0.5–2 MPa), completely avascular tissue (Cross et al., 2016). Moreover, most of the musculoskeletal lesions are allocated in the orthopedic tissue interfaces such as bone-cartilage, bone-tendon or bone-ligament, which naturally constitute a gradual transition from one tissue to the other, and consequently a gradual variation of tissue biochemical and mechanical characteristics (Cross et al., 2016). Therefore, engineering scaffolds for musculoskeletal regeneration is specially challenging and requires a minute control over material properties. Recent advances in materials design and production techniques permitted a fine control over scaffold microarchitecture and composition.

The matrix of tissues from the musculoskeletal system have a similar collagen rich composition, although they differ in its architectural assembly (Jiang et al., 2004). Mature cartilage matrix is highly hydrated and mostly contains collagen type II. When imaged by atomic force microscopy (AFM), cartilage showed fibrils of two sizes: wider fibrils of 180 ± 50 nm in diameter and a D-banding periodicity of 67.9 ± 1.2 nm, and thinner fibrils of 20 ± 10 nm diameter without distinguishable D-banding patterns (Zhu and Fang, 2012). In the case of tissues where collagen type I is more abundant, this fibrillar 3D meshwork structure is not observed, but micrometer size collagen fibril bundles are formed instead (Antipova and Orgel, 2010). In the tendon, collagen type I fibrils of 35–500 nm in diameter arranged forming bundles. Groups of these bundles form fascicles and fascicles get together to form a tendon. The alignment of collagen fibrils in the tendon was exclusively unidirectional and longitudinally oriented between muscle and bone providing tensile strength in this direction. A similar hierarchical arrangement of collagen fibrils is observed in ligaments (Woo and Levine, 1998) and bone. In bone, collagen type I aggregated into fibrils that regularly stack forming fibers leaving small gaps. These gaps are occupied by hydroxyapatite [Ca_10_(PO_4_)_6_(OH)_2_] like mineral spindles of 10–20 nm in length and 2–3 nm wide (Kane and Ma, 2013). In the case of muscle, the collagenous matrix (mostly type I and type III collagens) wraps muscle fibers thus mirroring their disposition and periodicity (Gillies and Lieber, 2011). When a muscle fiber is analyzed by AFM, the topography image showed the typical morphology of a sarcomere with irregularly spaced peaks for myosin filaments separated distances from 48 to 120 nm in agreement with previously reported X-ray diffraction results (Yamada et al., 2003; Li Y. et al., 2016).

**Figure 1:**
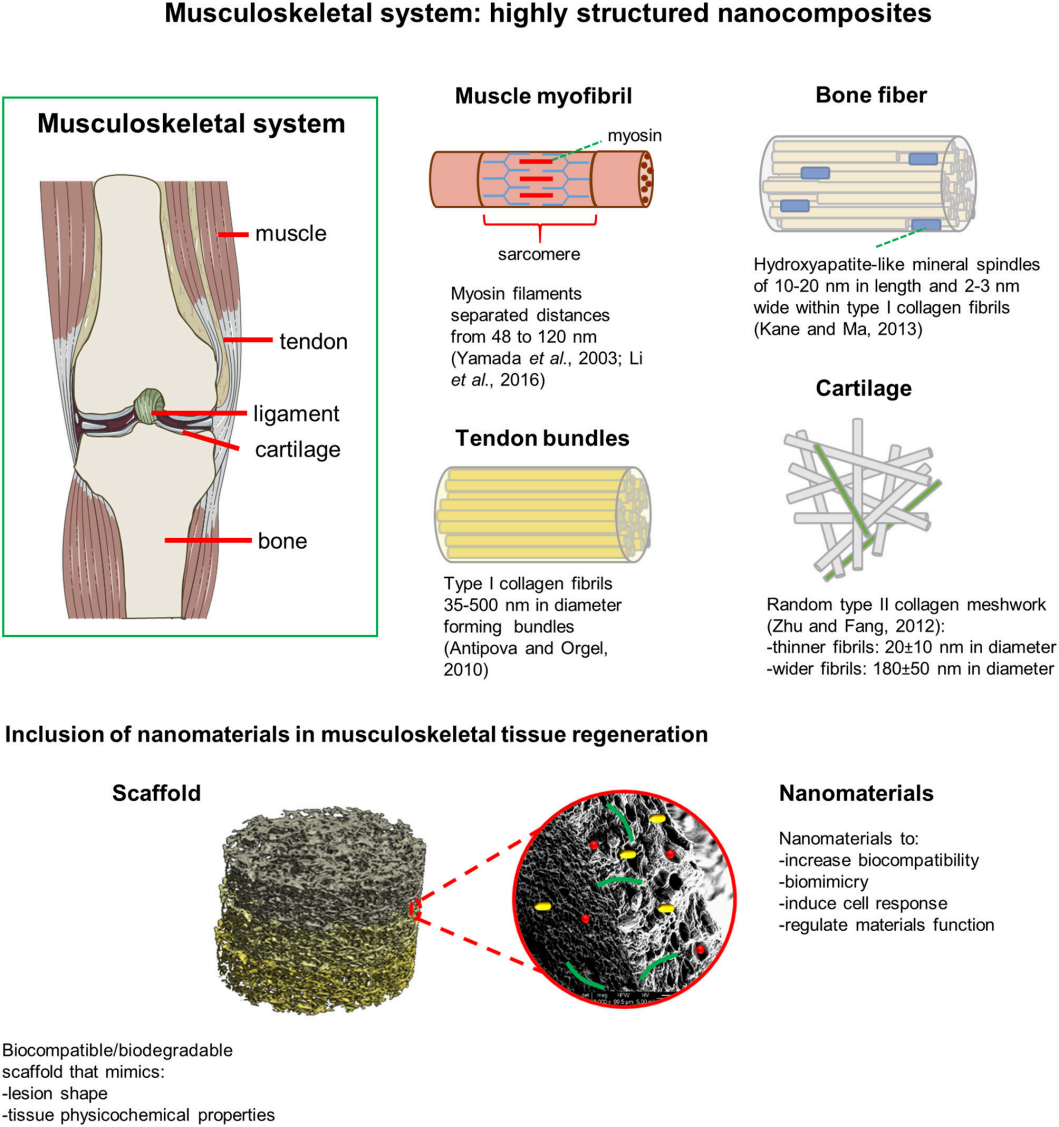
Musculoskeletal tissues can be considered as highly structured nanocomposites. Nanomaterials incorporated in the scaffold production better mimic tissue architecture, improve scaffold biocompatibility, regulate its mechanical and physicochemical properties, and direct cellular behavior.

Therefore, in their essential architecture, the musculoskeletal tissues can be considered highly structured nanocomposites (Egli and Luginbuehl, 2012). Accordingly, nanomaterials have been incorporated in the scaffold production to better mimic tissue architecture, improve material properties or direct cell behavior (Figure 1). The most recent advances in combining nanotechnology with 3D biomaterials engineering for musculoskeletal tissue regeneration are presented in this mini review.

## Nanofibrous scaffolds

Nanofibers have a distinctive high surface area for cell interaction and create a porous structure that facilitates the transfer of nutrients and cellular waste (Ma et al., 2013; Sankar et al., 2018). Self-assembled nanofibrous scaffolds have been produced to treat skeletal muscle defects (Cimenci et al., 2017). Self-assembled laminin mimetic peptide amphiphile nanofibers (LM/E-PA) with a diameter of around 6 nm and hundreds of nanometers in length, were shown to enhance *in vitro* myogenic differentiation and promote the effective myofibrillar regeneration after acute muscle injury in a rat model. In muscle fibers, cells are terminally differentiated, thus requiring the activation of satellite muscle progenitor cells for regeneration after injury. The extracellular matrix (ECM) protein laminin triggers the fusion of satellite cells with the existing fibers facilitating their regeneration. The bioactive peptide was designed containing the laminin epitope “IKVAV,” an alkyl tail and a β-sheet promoting VVAG sequence to self-assembly by hydrophobic collapse and form a nanofiber network morphologically like the natural ECM. Myogenesis was supported at the molecular level by LM/E-PA scaffolds through the early activation of satellite cells (Pax7 expression), significantly reducing the time required for the structural and functional repair of skeletal muscle of acute leg injury models in rat.

Nanofibrous scaffolds can also be produced by electrospinning. Electrospinning is a versatile and extensively used technique to produce nanofibrous structures, although with insufficient thickness and pore size for cell infiltration (Valizadeh and Mussa Farkhani, 2014). Nanofibrous scaffolds with fibers of hundreds of nanometers in length forming a high porosity mesh, and with enhanced mechanical properties for the regeneration of load-bearing bone defects, have been obtained by rolling microparticle-modified electrospun polycaprolactone /gelatin solutions (Hejazi and Mirzadeh, 2016). Coral microparticles were homogeneously added to the nanofibrous mat during electrospinning. Then, the mat was cut into strands and these strands were rolled up into a cylindrical shape. The presence of coral microparticles improved the open porosity within non-compact nanofibrous layers. It increased from 35.1 ± 0.5 % without microparticles to 67.1 ± 0.4 % maximum open porosity when 300 μm coral microparticles were included at 1:1 weight ratio to nanofibrous mat. Microparticles also modified the material elastic modulus, which increased from 3.547 ± 0.564 GPa without microparticles to 8.247 ± 1.476 GPa when 100 μm coral microparticles were included at 1:2 weight ratio. These values are comparable to those of natural cortical bone, which are around 16-23 GPa (Zioupos and Currey, 1998; Cross et al., 2016). Cultured MG-63 human bone osteosarcoma cells showed cell infiltration throughout the scaffolds with enhanced calcium deposition.

**Figure 2:**
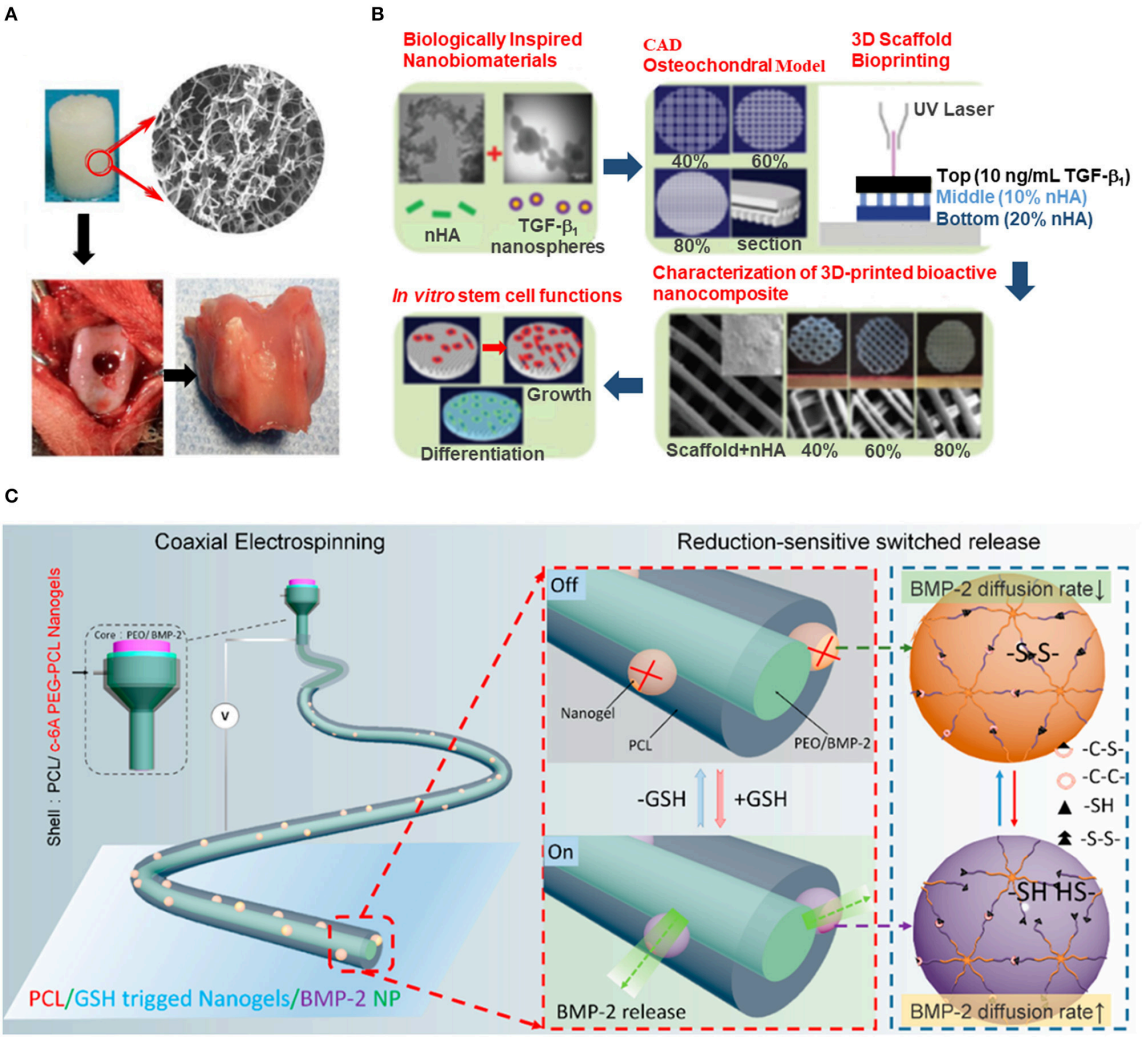
3D engineered nanomaterials for musculoskeletal tissue regeneration. **(A)** 3D scaffolds based on electrospun nanofibers of gelatin/PLA crosslinked with HA for osteochondral regeneration in rabbit model (adapted with permission from Chen et al., 2016, copyright, American Chemical Society). **(B)** Table-top SLA method to obtain porous hydrogel-based scaffolds that emulate the transitional nature of the osteochondral region by the inclusion of a gradient of nHA (adapted from Castro et al., 2015, with permission from The Royal Society of Chemistry). **(C)** Redox-responsive nanofibers allow the controlled BMP-2 release for bone regeneration in rat model (reprinted with permission from Gong et al., 2018, copyright American Chemical Society).

Mo and coworkers produced nanofibrous scaffolds from gelatin/poly(lactic acid) solutions by combining electrospinning and freeze drying techniques (Chen et al., 2016). The obtained 3D scaffolds were then heat-treated and cross-linked with hyaluronic acid for an upgraded cartilage regeneration. The scaffolds showed an excellent water absorption capacity and supported 60% compressive strain with a complete recovery of their initial shape once the compressing force was released. *In vitro* assays demonstrated that the cell cultures were viable and that the cultured chondrocytes could effectively penetrate inside the nanofibrous scaffolds. The scaffolds were successfully implanted in osteochondral defects produced in a rabbit model. Twelve weeks after implantation, treated defects were filled with uniform and well-integrated cartilage-like tissue (Figure 2A).

As shown in the examples presented, nanofibrous scaffolds recreate the essential architecture of the ECM, providing a more biomimetic environment for tissue regeneration with control in porosity and stiffness and the possibility of including bioactive compounds (Fernandes et al., 2016; Marino et al., 2017). Nevertheless, the level of accuracy in which the original tissue is reproduced by the scaffold is still limited.

## Nanomaterials in 3D printing

Strictly, 3D printing refers to computer-aided powder fusion-printing, where a jet of binder (solvent) is applied onto a bed of powder, fusing the particles of each layer in a layer-by-layer process (Moroni et al., 2018). Nevertheless, the term 3D printing in the literature has been broadened including other additive manufacturing and rapid prototyping techniques. They allow control on the design and production of scaffolds with complex structures and intricate geometries that better mimic tissue microarchitecture. Any structure feasible to be built by computer-aided design/computer-aided manufacturing (CAD/CAM) software can be fabricated through a layer-by-layer-based printing from slices of the digital model. Combined with high definition imaging, additive manufacturing techniques facilitated the production of native tissue-like scaffolds. They mimic the structure and function of the host tissue, which can be used as medical implants or as models in drug testing assays (Sears et al., 2016). Several additive manufacturing techniques adapted for tissue engineering applications have been developed (Sears et al., 2016; Nowicki et al., 2017).

Most printable materials are modified for an enhanced biocompatibility and cellular response (Jammalamadaka and Tappa, 2018). Particularly, the inclusion of nanomaterials during or after the fabrication process showed improved scaffold performance. Treatment with nanostructured hydroxyapatite (nHA) decreased pure iron (Fe) cytotoxicity, making Fe-based printed scaffolds suitable for bone regeneration with a significant improvement in their osteogenic capabilities (Yang et al., 2018). Metals, due to their mechanical properties, are highly convenient materials for prosthesis in load-bearing regions. Iron, as opposed to the more commonly used titanium, is biodegradable but presents low cytocompatibility due to the associated toxic iron release (IC50 = 18.4 mg/L). The repeated coating of printed Fe scaffolds with nHA nanorods of around 100 nm in diameter produced by hydrothermal treatment (Chen et al., 2006; Yang et al., 2018) structuraly mimicked the nano-spindle HA morphology of natural bone, significantly reduced iron release with increased cytocompatibility (2 mg/L after 4 coating cycles), and promoted osteogenesis of mesenchymal stem cells (MSCs).

Since bone is a highly vascularized tissue, scaffolds for bone tissue regeneration must present high interconnected porosity for nutrients and oxygen diffusion, while still preserving bone-like mechanical properties (Federovich et al., 2011). With additive manufacturing techniques, scaffolds with more uniform pore distribution can be produced with control on pore geometry, size, and interconnectivity. Zhang et al. developed a modified stereolithography (SLA) method for the fabrication of highly porous photocrosslinkable hydrogel scaffolds, which permitted the inclusion of bioinspired nanomaterials during the printing process (Castro et al., 2015). To emulate the transitional nature of the interfacing osteochondral region, a gradient of osteoconductive nHA nanorods of 80–100 nm in length and around 20 nm in diameter was printed within the subchondral bone layer. The chondrogenic transforming growth factor β1 was nanoencapsulated in core-shell poly(lactic-co-glycolic) acid nanospheres of 75 ± 17 nm in diameter for its sustained delivery, and was printed in the cartilage layer. Human MSCs *in vitro* adhesion, proliferation and osteochondral differentiation were highly increased on these graded nanocomposite osteochondral porous scaffolds (Figure 2B).

Mao and collaborators used Arg-Gly-Asp (RGD)-modified phage nanofibers (800 nm length per 6.6 nm wide) to fill in the pores of printed bone scaffolds for an improved vascularization in osteogenesis. The filamentous phage used in the study presents 3,000 copies of a helically ordered major coat protein. This protein has been genetically fused with RGD peptide leading to a high density ordered distribution of RGD on the virus capsid. The phage nanofibers were introduced together with chitosan (CH) in the pores of the bone-like scaffold. This activated endothelial cells migration and adhesion, inducing vascularization and promoting MSCs osteogenic fate *in vivo* (Wang et al., 2014).

Nanomaterials inclusion during or after printing has shown to improve scaffold biocompatibility, regulate the mechanical and physicochemical properties, and direct cellular performance. Therefore, the development of a additive manufacturing technique that allows direct nanoscale printing would be of interest to better mimic the nanostructure characteristics of the musculoskeletal tissue, which was still very preliminary emulated in the works cited.

### Nanomaterials in stimuli-responsive scaffolds

Stimuli-responsive materials are those capable of modifying one or more of their properties when exposed to an external stimulus. As in living systems, the ability of materials to act in response to external signals renders improved adaptation to the surrounding environment. The material can respond to light, pH, temperature, applied mechanical force, electrical and magnetic fields or chemicals among others (Stuart et al., 2010; Khan and Tanaka, 2018). The combination of stimuli-responsiveness with nanotechnology enabled the fine tuning of material properties for an increased performance in tissue engineering applications (Skorb and Andreeva, 2013).

Stimuli-responsive materials allow for the controlled release of growth factors, which can be inactive until the stimuli triggers their release, thus avoiding their early inactivation. Magneto-responsive nanogels have been developed for the controlled release of the osteogenic growth factor bone morphogenetic protein-2 (BMP-2) and promote the viability of MG-63 cells (Fan et al., 2014). CH and heparin, functionalized with adenine and thymine respectively, were used as the nanogel precursors in a nucleobase pairing self-assembly process, mediated by hydrogen bonding. Magneto-responsiveness was obtained by encapsulating super-para-magnetic iron oxide (Fe_3_O_4_) nanoparticles of around 15 nm in diameter into the nanogel spheres of sizes among 60–160 nm. BMP-2 was adsorbed from solution on the nanogels and retained with a high loading efficiency through heparin binding, which also protected BMP-2 from proteolytic degradation.

*In vivo* mandibular bone reconstruction has been addressed by redox-responsive nanofiber-based implants produced by coaxial electrospinning (Gong et al., 2018). BMP-2 was loaded in the 220–340 nm in length nanofiber inner core of poly(ethylene oxide). The redox-switched release of BMP-2 was achieved in response of variable concentrations of glutathione (GSH), which caused the reversible breakage of the disulfide bonds present in the outer shell of the nanofibers. This outer shell was composed by poly(ε-caprolactone) (PCL) and the redox-responsive c-6A poly(ethylene glycol)-PCL nanogel of 96 ± 3 nm in diameter, which acted as an on-off switchable valve in response to GSH concentration. Permeable nanochannels on the gel were generated upon the breakage of disulfide bonds, allowing BMP-2 to be released (Figure 2C). The implanted redox-responsive nanofibers were shown to promote *in vitro* osteogenesis and *in vivo* mandible defect repair in rat model.

Stupp and coworkers developed injectable liquid crystalline nanofibrous scaffolds formed by peptide amphiphile that encapsulate cells and growth factors within a muscle-like aligned environment for muscle progenitor cell transplantation *in vivo* (Sleep et al., 2017). An aliphatic palmitoyl tail covalently linked to a peptide sequence of 6 to 9 aminoacids self-assembles into liquid crystal aligned nanofibers of 150–700 nm in length, upon extrusion into physiological calcium concentrations in culture medium or upon injection *in vivo*. Such structures presented stiffness values that closely mimic that of the skeletal muscle (5–40 kPa elastic modulus; Gilbert et al., 2010). C2C12 mouse skeletal muscle progenitor cells, fetal bovine serum and basic fibroblast growth factor were encapsulated into the aligned nanofibrous scaffolds. Good cell viability, proliferation, alignment and maturation results were obtained *in vitro* and good cell engraftment was observed *in vivo* in injured muscles in mice.

Nanostructured stimuli-responsive hydrogels (SRHs) in which sol-gel transition can be induced have numerous applications in cartilage repair. They effectively mimic tissue mechanical properties and the nanostructured nature of the cartilage ECM and, when gelation occurs near the body temperature, they can be injected into the lesion site using minimally invasive surgery (Eslahi et al., 2016a; Kim and Matsunaga, 2017). Bonakdar and coworkers obtained good cytocompatibility and cell adhesion for chondrocytes encapsulated in thermo-responsive hydrogel composites with superior viscoelastic properties due to the inclusion of silicate nanodiscs of 20–30 nm in diameter and 1–2 nm thickness (Eslahi et al., 2016b). Thermosensitive Pluronic (Pl) hydrogel conjugated with CH to mimic cartilage ECM (PlCH) was crosslinked with keratin (an ECM fibrous protein) using biocompatible genipin as crosslinking agent. Silicate nanodiscs were incorporated in the mixture during the crosslinking process to enhance hydrogel stability.

Nanoparticles included in SRHs can sufficiently increase hydrogel stiffness for bone tissue engineering applications. Qian and coworkers produced injectable thermo-sensitive hydrogels with nHA for bone regeneration of calvarial defects in a rabbit model (Fu et al., 2012). Thermo-responsive hydrogel was prepared using the triblock copolymer poly(ethylene glycol)-poly(ε-caprolactone)-poly(ethylene glycol) (PECE). The hydrogel was modified with 10% collagen and 30% nHA of 20–40 nm in diameter and 80–120 nm length to mimic bone ECM and to improve hydrogel bioactivity and increase rigidity, while still preserving the gelation temperature near 37°C (Fu et al., 2009). Radiological and histological analysis on rabbit calvarial defects treated with the injectable PECE/Collagen/n-HA hydrogel composite showed good guided bone regeneration compared to the self-repair process.

## Conclusions

The inner complexity and diversity of tissues integrating the musculoskeletal system makes tissue engineering particularly challenging in this field. This mini-review summarizes the most recent advances in materials design and production techniques that, combined with nanotechnology, permitted a fine control over scaffold micro- and nano-architecture, composition and behavior for a better tissue integration. It is expected that engineered artificial tissues for musculoskeletal regeneration and for tissue repair in general will continue to evolve. additive manufacturing strategies could be merged with stimuli-responsive materials as predicted by Khademhosseini et al. toward four-dimensional (4D) bioprinting (Li Y. C. et al., 2016). They certainly will harness the inclusion of nanotechnology to broaden the spectrum of these new materials' capabilities.

## Author contributions

AL wrote the manuscript with contributions from IC, AG-L, and JS.

### Conflict of interest statement

The authors declare that the research was conducted in the absence of any commercial or financial relationships that could be construed as a potential conflict of interest.

## References

[B1] Antipova O. Orgel J. P. (2010). *In situ* D-periodic molecular structure of type II collagen. J. Biol. Chem. 285, 7087–7096. 10.1074/jbc.M109.060400 20056598 PMC2844158

[B2] Castro N. J. O'Brien J. Zhang L. G. (2015). Integrating biologically inspired nanomaterials and table-top stereolithography for 3D printed biomimetic osteochondral scaffolds. Nanoscale 7, 14010–14022. 10.1039/C5NR03425F 26234364 PMC4537413

[B3] Chen H. Tang Z. Liu J. Sun K. Chang S. -R. Peters M. C. (2006). Acellular synthesis of a human enamel-like microstructure. Adv. Mater. 18, 1846–1851. 10.1002/adma.200502401

[B4] Chen W. Chen S. Morsi Y. El-Hamsshary H. El-Newhy M. Fan C. (2016). Superabsorbent 3D scaffold based on electrospun nanofibers for cartilage tissue engineering. ACS Appl. Mater. Interfaces 8, 24415–24425. 10.1021/acsami.6b06825 27559926

[B5] Cross L. M. Thakur A. Jalili N. A. Detamore M. Gaharwar A. K. (2016). Nanoengineered biomaterials for repair and regeneration of orthopedic tissue interfaces. Acta Biomater. 42, 2–17. 10.1016/j.actbio.2016.06.023 27326917

[B6] Egli R. J. Luginbuehl R. (2012). Tissue engineering–nanomaterials in the musculoskeletal system. Swiss Med Wkly. 142:w13647. 10.4414/smw.2012.13647 22850986

[B7] Eren Cimenci C. Uzunalli G. Uysal O. Yergoz F. Karaca Umay E. Guler M. O.. (2017). Laminin mimetic peptide nanofibers regenerate acute muscle defect. Acta Biomater. 60, 190–200. 10.1016/j.actbio.2017.07.010 28690008

[B8] Eslahi N. Abdorahim M. Simchi A. (2016a). Smart polymeric hydrogels for cartilage tissue engineering: a review on the chemistry and biological functions. Biomacromolecules 17, 3441–3463. 10.1021/acs.biomac.6b01235 27775329

[B9] Eslahi N. Simchi A. Mehrjoo M. Shokrgozar M. A. Bonakdar S. (2016b). Hybrid cross-linked hydrogels based on fibrous protein/block copolymers and layered silicate nanoparticles: tunable thermosensitivity, biodegradability and mechanical durability. RSC Adv. 6, 62944–62957. 10.1039/C6RA08563F

[B10] Fan M. Yan J. Tan H. Miao Y. Hu X. (2014). Magnetic biopolymer nanogels via biological assembly for vectoring delivery of biopharmaceuticals. J. Mater. Chem. B. 2, 8399–8405. 10.1039/C4TB01106F 32262010

[B11] Federovich N. E. Kuipers E. Gawlitta D. Dhert W. J. Alblas J. (2011). Scaffold porosity and oxygenation of printed hydrogel constructs affect functionality of embedded osteogenic progenitors. Tissue Eng. A 17, 2473–2486. 10.1089/ten.tea.2011.0001 21599540

[B12] Fernandes J. S. Gentile P. Martins M. Neves N. M. Miller C. Crawford A.. (2016). Reinforcement of poly-L-lactic acid electrospun membranes with strontium borosilicate bioactive glasses for bone tissue engineering. Acta Biomaterialia 44, 168–177. 10.1016/j.actbio.2016.08.042 27554018

[B13] Fu S. Z. Guo G. Gong C. Y. Zeng S. Liang H. Luo F.. (2009). Injectable biodegradable thermosensitive hydrogel composite for orthopedic tissue engineering, 1. Preparation and characterization of nanohydroxyapatite/poly(ethylene glycol)-poly(ε-caprolactone)-poly(ethylene glycol) hydrogel nanocomposites. J. Phys. Chem. B. 113, 16518–16525. 10.1021/jp907974d 19947637

[B14] Fu S. Z. Ni P. Y. Wang B. Y. Chu B. Y. Zheng L. Luo F. (2012). Injectable and thermos-sensitive PEG-PCL-PEG copolymer/collagen/n-HA hydrogel composite for guided bone regeneration. Biomaterials 33, 4801–4809. 10.1016/j.biomaterials.2012.03.040 22463934

[B15] Gilbert P. M. Havenstrite K. L. Magnusson K. E. Sacco A. Leonardi N. A. Kraft P.. (2010). Substrate elasticity regulates skeletal muscle stem cell self-renewal in culture. Science 329, 1078–1081. 10.1126/science.1191035 20647425 PMC2929271

[B16] Gillies A. R. Lieber R. L. (2011). Structure and function of the skeletal muscle extracellular matrix. Muscle Nerve 44, 318–331. 10.1002/mus.22094 21949456 PMC3177172

[B17] Gong T. Liu T. Zhang L. Ye W. Guo X. Wang L. (2018). Design redox-sensitive drug-loaded nanofibers for bone reconstruction. ACS Biomater. Sci. Eng. 4, 240–247. 10.1021/acsbiomaterials.7b00827 33418691

[B18] Hejazi F. Mirzadeh H. (2016). Roll-designed 3D nanofibrous scaffold suitable for the regeneration of load bearing bone defects. Prog. Biomater. 5, 199–211. 10.1007/s40204-016-0058-2 27995587 PMC5301453

[B19] Jammalamadaka U. Tappa K. (2018). Recent advances in biomaterials for 3D printing and tissue engineering. J. Funct. Biomater. 9, 22–36. 10.3390/jfb9010022 29494503 PMC5872108

[B20] Jiang F. Horber H. Howard J. Muller D. J. (2004). Assembly of collagen into microribbons: effects of pH and electrolytes. J. Struct. Biol. 148, 268–278. 10.1016/j.jsb.2004.07.001 15522775

[B21] Kane R. Ma P. X. (2013). Mimicking the nanostructure of bone matrix to regenerate bone. Mater. Today 16, 418–423. 10.1016/j.mattod.2013.11.001 24688283 PMC3968917

[B22] Khan F. Tanaka M. (2018). Designing smart biomaterials for tissue engineering. Int. J. Mol. Sci. 19, 17–31. 10.3390/ijms19010017 29267207 PMC5795968

[B23] Kim Y. -J. Matsunaga Y. T. (2017). Thermo-responsive polymers and their application as smart biomaterials. J. Mater. Chem. B. 5, 4307–4321. 10.1039/C7TB00157F 32263961

[B24] Li Y. Lang P. Linke W. A. (2016). Titin stiffness modifies the force generating region of muscle sarcomeres. Sci. Rep. 6:24492. 10.1038/srep24492 27079135 PMC4832248

[B25] Li Y. C. Zhang Y. S. Akpek A. Shin S. R. Khademhosseini A. (2016). 4D bioprinting: the next-generation technology for biofabrication enabled by stimuli-responsive materials. Biofabrication 9, 012001–0120016. 10.1088/1758-5090/9/1/012001 27910820

[B26] Ma B. Xie J. Jiang J. Shuler F. D. Bartlett D. E. (2013). Rational design of nanofiber scaffolds for orthopedic tissue repair and regeneration. Nanomedicine 8, 1459–1481. 10.2217/nnm.13.132 23987110 PMC3875778

[B27] Marino A. Tonda-Turo C. De Pasquale D. Ruini F. Genchi G. Nitti S.. (2017). Gelatin/nanoceria nanocomposite fibers as antioxidant scaffolds for neuronal regeneration. Biochim. Biophys. Acta 1861, 386–395. 10.1016/j.bbagen.2016.11.022 27864151

[B28] Moroni L. Boland T. Burdick J. A. De Maria C. Derby B. Forgacs G. (2018). Biofabrication: a guide to technology and terminology. Trends Biotechnol. 36, 384–402. 10.1016/j.tibtech.2017.10.015 29137814

[B29] Nowicki M. Castro N. J. Rao R. Plesniak M. Zhang L. G. (2017). Integrating three-dimensional printing and nanotechnology for musculoskeletal regeneration. Nanotechnology 28, 382001–382014. 10.1088/1361-6528/aa8351 28762957 PMC5612478

[B30] Sankar S. Sharma C. S. Rath S. N. Ramakrishna S. (2018). Electrospun nanofibers to mimic natural hierarchical structure of tissues: application in musculoskeletal regeneration. J. Tissue. Eng. Regen. Med. 12, e604–e619. 10.1002/term.2335 27686061

[B31] Sears N. A. Seshadri D. R. Dhavalikar P. S. Cosgriff-Hernandez E. (2016). A review of three-dimensional printing in tissue engineering. Tissue Eng. Part B Rev. 22, 298–310. 10.1089/ten.teb.2015.0464 26857350

[B32] Skorb E. V. Andreeva D. V. (2013). Surface nanoarchitecture for bio-applications: self-regulating intelligent interfaces. Adv. Funct. Mater. 23, 4483–4506. 10.1002/adfm.201203884

[B33] Sleep E. Cosgrove B. D. McClendon M. T. Preslar A. T. Chen C. H. Sangji M. H. (2017). Injectable biomimetic liquid crystalline scaffolds enhance muscle stem cell transplantation. Proc. Natl. Acad. Sci. U.S.A. 114, E7919–E7928. 10.1073/pnas.1708142114 28874575 PMC5617293

[B34] Smith B. D. Grande D. A. (2015). The current state of scaffolds for musculoskeletal regenerative applications. Nat. Rev. Rheumatol. 11, 213–222. 10.1038/nrrheum.2015.27 25776947

[B35] Storheim K. Zwart J.-A. (2014). Musuloskeletal disorders and the global burden of disease study. Ann. Rheum. Dis. 73, 949–950. 10.1136/annrheumdis-2014-205327 24790065

[B36] Stuart M. A. Huck W. T. Genzer J. Müller M. Ober C. Stamm M.. (2010). Emerging applications of stimuli-responsive polymer materials. Nat. Mater. 9, 101–113. 10.1038/nmat2614 20094081

[B37] Valizadeh A. Mussa Farkhani S. (2014). Electrospinning and electrospun nanofibers. IET Nanobiotechnol. 8, 83–92. 10.1049/iet-nbt.2012.0040 25014079

[B38] Wang J. Yang M. Zhu Y. Wang L. Tomsia A. P. Mao C. (2014). Phage nanofibers induce vascularized osteogenesis in 3D printed bone scaffolds. Adv. Mater. 26, 4961–4966. 10.1002/adma.201400154 24711251 PMC4122615

[B39] Woo S. L.-Y. Levine R. E. (1998). Ligament, tendon and facia, in Handbook of Biomaterial Properties, eds Black J. Hastings G. (Boston, MA: Springer), 59–65. 10.1007/978-1-4615-5801-9_6

[B40] Yamada T. Kunioka Y. Wakayama J. Aimi M. Noguchi Y. S. Akiyama N.. (2003). Molecular organizations of myofibrils of skeletal muscle studied by atomic force microscopy. Adv. Exp. Med. Biol. 538, 285–294. 10.1007/978-1-4419-9029-7_27 15098676

[B41] Yang C. Huan Z. Wang X. Wu C. Chang J. (2018). 3D printed Fe scaffolds with HA nanocoating for bone regeneration. ACS Biomater. Sci. Eng. 4, 608–616. 10.1021/acsbiomaterials.7b00885 33418749

[B42] Zhu P. Fang M. (2012). Nano-morphology of cartilage in hydrated and dehydrated conditions revealed by atomic force microscopy. J. Phys. Chem. Biophys. 2:1000106 10.4172/2161-0398.1000106

[B43] Zioupos P. Currey J. D. (1998). Changes in the stiffness, strength, and toughness of human cortical bone with age. Bone 22, 57–66. 10.1016/S8756-3282(97)00228-7 9437514

